# Temporomandibular Joint Ankylosis: Anesthetic Challenge

**DOI:** 10.7759/cureus.54379

**Published:** 2024-02-17

**Authors:** Nikhil Bhalerao, Saely Paunikar, Dnyanshree Wanjari, Sanjot Ninave

**Affiliations:** 1 Anesthesiology, Datta Meghe Institute of Higher Education and Research, Wardha, IND

**Keywords:** nasal intubation, paediatric airway, fibreoptic bronchoscope, temporomandibular joint (tmj) disorders, difficult airway management

## Abstract

Temporomandibular joint ankylosis cases serve as a challenge for both surgeons and anesthesiologists possibly due to the unavailability of resources in remote locations. Distressing issues brought on by its ankylosis include functional as well as esthetic issues such as considerable difficulties in managing the airway, especially in children because of the physiology and structure of their airways being different. Fiberoptic bronchoscopy (FOB) has a well-established role in patients with difficult airways, but it is especially challenging in pediatric patients because of their lack of cooperation and diminished lung reserve. Techniques used to secure airways in adults may not be ideal for children and sometimes dedicated equipment may not be available. Here we present a case of a 14-year-old boy with temporomandibular joint (TMJ) ankylosis. This study aimed to describe the difficulties experienced in managing his airway.

## Introduction

Greek terminology meaning "stiff joint" is the basis of the word ankylosis. Temporomandibular joint (TMJ) ankylosis can be bony or fibrous, true (intraarticular) or false (extraarticular) [1]. Children who have TMJ ankylosis may develop mandibular retrognathism, which can lead to functional and esthetic deficiencies [2]. This can result in limited movement of the jaw and difficulty with activities, such as speaking, eating, and opening the mouth. In addition to having symptoms of severe obstructive sleep apnea (OSA), patients with bilateral TMJ ankylosis frequently experience micrognathia and retrognathia. TMJ ankylosis can arise from various causes, including but not limited to trauma, arthritis, infection, prior TMJ surgery, congenital abnormalities, idiopathic variables, and iatrogenic causes [3].

Treatment for TMJ ankylosis typically involves surgical intervention, which may include joint reconstruction, joint replacement, or the removal of fused tissue or bone. Physical therapy and medications may also be used to manage symptoms and improve jaw function. Early intervention is important to prevent further damage and improve outcomes. There are following three options for intubating a patient with minimal or nonexistent mouth opening: blind nasal intubation, retrograde intubation using cricothyroid puncture, or fiberoptic intubation. Because children's physiology and airway structure differ from adults', managing airway symptoms of temporomandibular joint (TMJ) ankylosis can be particularly difficult [1]. Because of the reduced area in the airways, extreme caution must be used when attempting fiberoptic bronchoscopy in pediatric patients. The decision is based on the patient's age, clinical situation, level of cooperation, level of experience, and equipment availability.

## Case presentation

A 14-year-old, 11 kg male patient, who underwent surgery for bilateral TMJ ankylosis with a distractor device in situ, presented at the hospital for further management. As narrated by the parents, the patient had inadequate mouth opening since birth. Patient successfully underwent surgery for mandibular distraction with a device in situ eight months back and had achieved a mouth opening of 25 mm post-surgery. The patient was not diagnosed with any other comorbidities. Upon preanesthetic check-up, the patient was conscious, oriented, and vitally stable with oral examination revealing mouth opening of 25 mm and midline deviated towards the right. Considering difficult airway, the plan of anesthesia was fibreoptic-guided nasal intubation. Emergency cricothyroidotomy and tracheostomy sets were kept ready in case of emergency. Thirty minutes before moving to the operating room (OR), nebulization with two milliliters of 4% lignocaine and the application of xylometazoline nasal drops in both nostrils was done.

**Figure 1:**
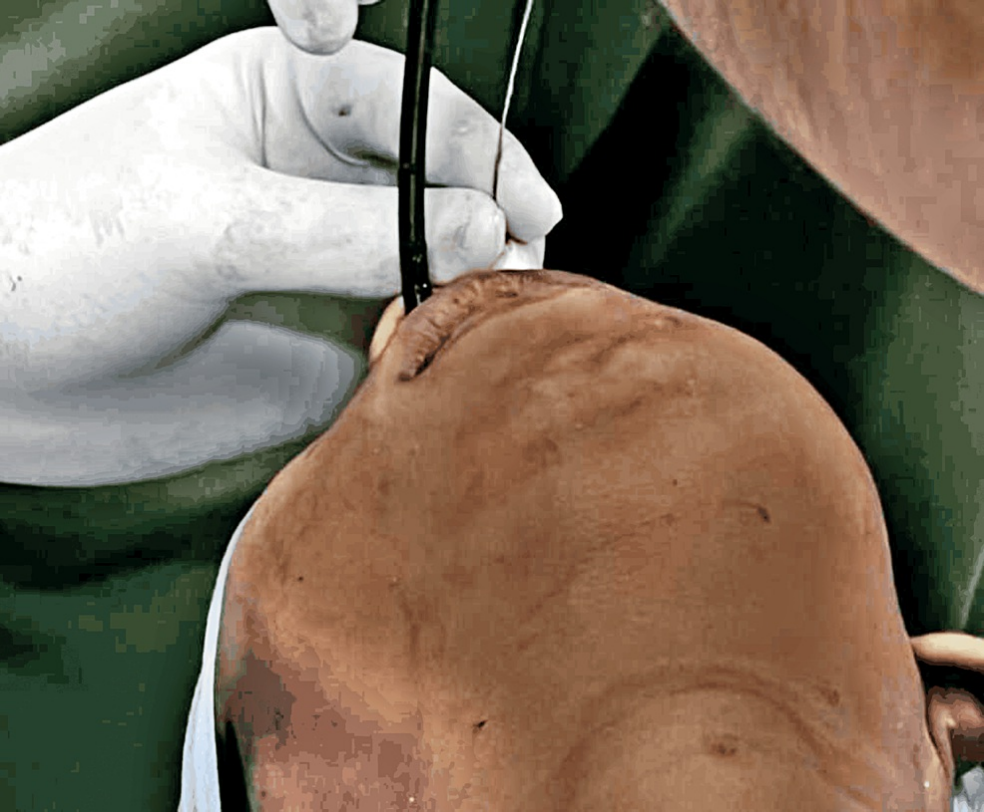
Bronchoscope and guide wire in two different nostrils.

**Figure 2:**
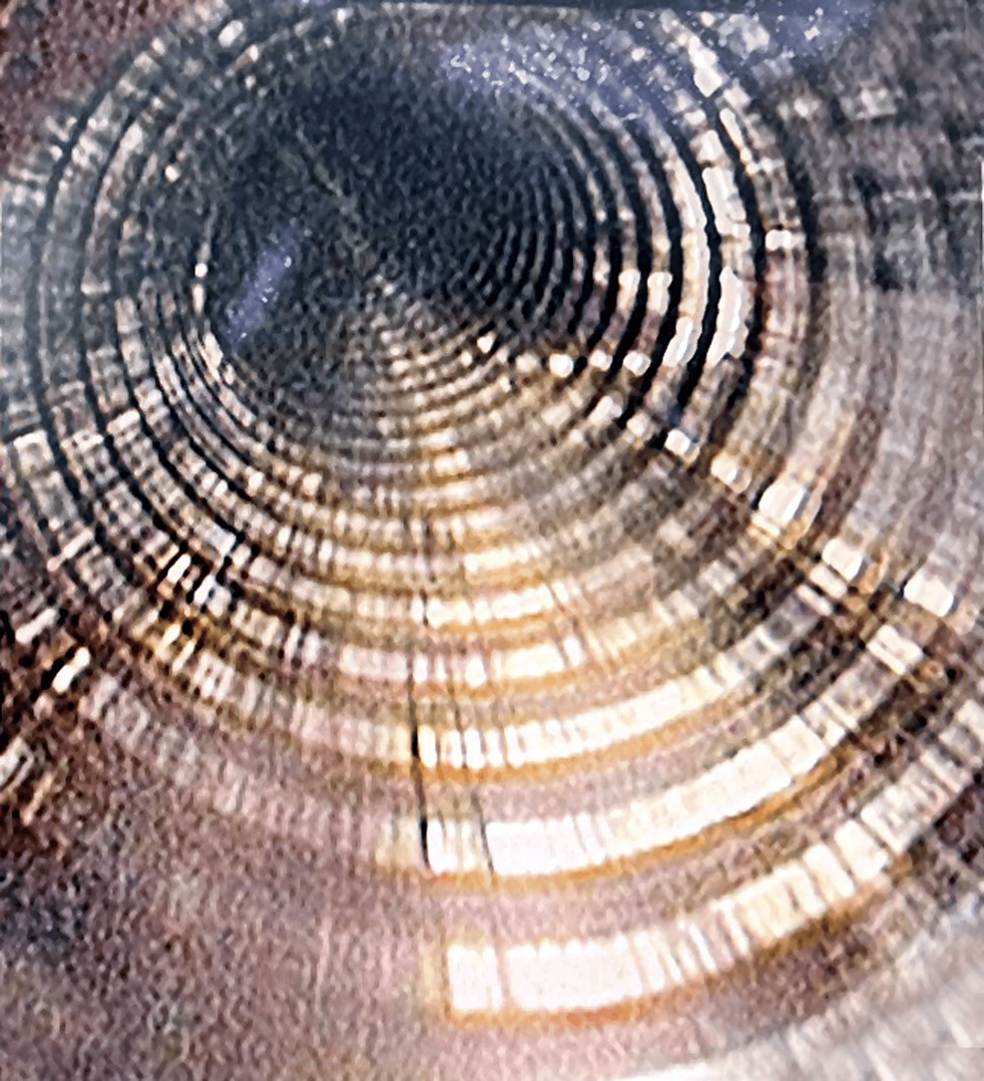
Endotracheal tube (ETT) advanced in trachea.

Attached were the necessary monitoring devices (capnograph, noninvasive blood pressure, electrocardiography, and pulse oximeter). Premedication in the OR was done with injection of glycopyrrolate 0.1 mg, midazolam 1.25 mg, injection of fentanyl 25 mcg followed by injection of ketamine 40 mg IV. Adequate sedation was achieved and a fibreoptic bronchoscope with adequate endotracheal tube (ETT) size no. according to the following formula was advanced through the right nostril: age/4 + 3.5=6.5 mm tube. However, this attempt was unsuccessful as the tube could not be advanced beyond nostril. Due to the unavailability of pediatric bronchoscope in our hospital (Acharya Vinoba Bhave Rural Hospital) setting, ETT no. 6 mm could not be used as we had a standard adult-size bronchoscope (6 mm). Hence with the bronchoscope in the left nostril (a glottic view), a guidewire was inserted through the right nostril, and ETT no. 6 was glided over it (Figure 1). After the tube was visible in the glottic aperture, ETT was softly advanced (Figure 2).

Both the end-tidal CO_2_ trace on the capnograph and auscultation were used for confirmation and an injection of vecuronium 3 mg was given. O_2_, N_2_O, sevoflurane, vecuronium, and volume control modes of ventilation were used to maintain anesthesia. The surgeon verified the mouth opening with a 30 mm interincisor space prior to extubation. Upon completion of the procedure, the patient was smoothly reversed and extubated.

## Discussion

Temporomandibular joint (TMJ) ankylosis is a condition in which there is a fusion or fixation of the joint between the lower jawbone (mandible) and the skull (temporal bone). Because of their decreased mouth opening, reduced jaw protrusions, and possible mandibular hypoplasia, patients with TMJ ankylosis are projected to have difficult intubation as well as mask ventilation [3]. Enhancing the patient's mandibular function, addressing any related facial deformities, reducing pain, and preventing ankylosis are the objectives of care for TMJ ankyloses [4]. It is stated that maintaining a patent airway is the anesthesiologist's primary concern [5]. With limited mouth opening, the potential options are retrograde intubation, tracheostomy, and nasal intubation with either blind or fiberoptic assistance. Despite being largely safe, nasotracheal intubations (NTIs) nevertheless carry the risk of significant side effects, including trauma and nasopharyngeal hemorrhage [6].

The gold standard is a fiberoptic video laryngoscope since blind nasal intubation can damage the middle or inferior epistaxis, nasal mucosal damage, infection, and turbinate. Patient's cooperation, local blocks for the laryngeal nerves, and topical anesthetic for the upper airway are required during awake intubation [7]. When considering pediatric patients for anesthesia, it is important to keep in mind the physiological and anatomical variations that exist between an adult and pediatric patient [8]. For pediatric patients, awake fiberoptic intubation is rarely possible; instead, deep sedation or general anesthesia may be necessary [8]. It may become impossible to intubate a child if repeated efforts at intubation develop airway edema and stress to the airway. There should only be three or four attempts at intubation at most [9]. When working with a complicated airway, fiberoptic nasotracheal intubation is performed by rail loading the ETT via the fiberoptic scope as a single assembly and maintenance of anesthesia, either with an ETT inserted from the opposite nostril or a nasopharyngeal airway [10]. There are a number of complications related to pediatric tracheostomy. Although the death rate from tracheostomies is quite low, the total death rate from tracheostomized patients is not insignificant [11]. Tube obstruction is the most frequent tracheostomy-related cause of death in pediatric patients, followed by tube misplacement and unintentional decannulation [12]. Because an appropriate-sized bronchoscope was not available, we had to improvise our procedure. The only limitation to this procedure was a failure to achieve a secured airway leading to abandonment of the procedure or tracheostomy which has its own adverse outcomes. In our case, tracheostomy was avoided as the patient didn’t require prolonged intubation and other techniques had not been implemented. Retrograde intubation is a challenging procedure that is rarely used on a young patient who has restricted mouth opening. Since tracheostomy is an intrusive treatment with associated morbidity, it is often saved for emergency situations. Dhulkhed in 2008 presented a case of TMJ ankylosis with successful retrograde intubation avoiding the demerits of nasal intubation of airway trauma and bleeding [13]. In a 2017 study, Pokhriyal et al. compared the hemodynamic response and success rate of awake retrograde endotracheal intubation (REI) and fiberoptic bronchoscope (FOB) assisted endotracheal intubation in difficult airway situations. They found that the maximum increase in heart rate and mean arterial pressure (MAP) occurred at the end of intubation in the REI group compared to the FOB group, while the FOB group's intubation took less time and had a higher success rate than the REI group [14]. In their retrospective observational analysis, Kriege et al. found that tracheal intubation performed while awake was linked to a decreased rate of serious adverse outcomes compared to tracheal intubation performed while anesthetized [15]. The anesthesia team's closed-loop operation has a positive impact on the outcome. The prompt thinking of the anesthesia team made this case successful, barring any complications. It is always ideal to extubate a patient when they are completely conscious, with adequate, spontaneous, and regular breathing [8]. Shetty et al. performed a five-year audit in a university hospital on anesthetic management of temporomandibular joint ankylosis in pediatrics and concluded that the best option for intubation in cases of TMJ ankylosis was still awake fiberoptic intubation or with light sedation, despite the fact that different anesthesiologists used varied procedures [1].

## Conclusions

Pediatric difficult airway management is particularly challenging and demands knowledge, preparation, and the ability to navigate any complications that may arise from the attempted intubation. Compared to other types of intubation, as this improvised technique showed minimal adverse outcomes, this patient was successfully intubated making the best use of available resources.
